# Identifying mechanisms of change in a conversation therapy for aphasia using behaviour change theory and qualitative methods

**DOI:** 10.1111/1460-6984.12279

**Published:** 2016-11-23

**Authors:** Fiona M. Johnson, Wendy Best, Firle Christina Beckley, Jane Maxim, Suzanne Beeke

**Affiliations:** ^1^ University College London Division of Psychology & Language Sciences London WC1E 6BT UK; ^2^ Homerton University Hospital NHS Foundation Trust

**Keywords:** conversation therapy, aphasia, behaviour change

## Abstract

**Background:**

Conversation therapy for aphasia is a complex intervention comprising multiple components and targeting multiple outcomes. UK Medical Research Council (MRC) guidelines published in 2008 recommend that in addition to measuring the outcomes of complex interventions, evaluation should seek to clarify how such outcomes are produced, including identifying the hypothesized mechanisms of change.

**Aims:**

To identify mechanisms of change within a conversation therapy for people with aphasia and their partners. Using qualitative methods, the study draws on behaviour change theory to understand how and why participants make changes in conversation during and after therapy.

**Methods & Procedures:**

Data were derived from 16 participants (eight people with aphasia; eight conversation partners) who were recruited to the Better Conversations with Aphasia research project and took part in an eight session conversation therapy programme. The dataset consists of in‐therapy discussions and post‐therapy interviews, which are analysed using Framework Analysis.

**Outcomes & Results:**

Seven mechanisms of conversational behaviour change are identified and linked to theory. These show how therapy can activate changes to speakers’ skills and motivation for using specific behaviours, and to the conversational opportunities available for strategy use.

**Conclusions & Implications:**

These clinically relevant findings offer guidance about the processes involved in producing behavioural change via conversation therapy. A distinction is made between the process involved in motivating change and that involved in embedding change. Differences are also noted between the process engaged in reducing unhelpful behaviour and that supporting new uses of compensatory strategies. Findings are expected to have benefits for those seeking to replicate therapy's core processes both in clinical practice and in future research.

**What this paper adds**

**What is already known on the subject?**

The literature on conversation therapy for aphasia emphasizes increased awareness of conversational patterns to promote change. However, it is not clear how this process maps onto the specific behavioural outcomes that are typically targeted and measured by therapy.

**What this paper adds**

This paper offers a systematic account of change via therapy and examines the evidence for the mechanisms of conversational behavioural change within a specific programme. The difference between change that involves reducing or terminating previous behaviour, and change that involves developing new behaviour is clarified. This paper identifies seven potential mechanisms of conversational behaviour change, and explores their links to explanatory theory and experimental evidence. It introduces the COM‐B model of behaviour and illustrates how it can be applied to conversation therapy to further our understanding of how therapy produces its outcomes.

## Introduction

Intervention research is typically focused on the question: Does this treatment work? However, outcome‐focused evidence provides surprisingly little information about *how* treatment works, and the therapeutic processes that are responsible for producing change. The umbrella term ‘conversation therapy’ covers a wide range of treatment procedures and approaches (Simmons‐Mackie *et al*. 2014). Explanatory theory able to link therapy's core processes to its outcomes is needed in order to support replication in clinical practice (Kagan *et al*. 2010, Whyte *et al*. 2014). The MRC (2008) guidelines on designing and evaluating complex interventions propose that developing a ‘theory of change’ should be a vital component of intervention research, and that the intervention process is routinely evaluated alongside outcomes. Viewing conversation therapy for aphasia as a ‘complex intervention’ highlights the need to delineate better the core mechanisms that enable or trigger changes to the way speakers manage aphasia in conversation. This study, therefore, aims to systematically investigate therapeutic mechanisms of conversational change by exploring participants’ experience within a programme known as Better Conversations with Aphasia (BCA) (Beeke *et al*. 2013b). It will focus specifically on the primary outcomes targeted by BCA, i.e., the adoption of compensatory strategies, and the reduction or termination of conversational barriers. This introduction provides further detail on BCA, as well as background on behaviour change theory before outlining the study's specific research objectives.

### Better Conversations with Aphasia (BCA)

Conversation therapies for aphasia aim to maximize the social participation and communicative success of people with aphasia within everyday interaction. Many focus on the skills of key conversation partners (CPs) at managing the impact of aphasia on conversation, whilst others work jointly with the person with aphasia (PWA) and their CP. For a review of the field, see Simmons‐Mackie *et al*. (2014). BCA is a development of the CP training programme ‘Supporting Partners of People with Aphasia in Relationships and Conversation’ (SPPARC) (Lock *et al*. 2001). It includes the PWA in therapy, and originally had a specific focus on non‐fluent, agrammatic aphasia. The programme covers conversational issues of general relevance in aphasia however, and has been adopted by clinicians for use with a wider population. As in SPPARC, BCA's theoretical roots lie with conversation analysis (CA) (Hutchby and Wooffitt 2008), a technique used to explore patterns of breakdown and resolution within conversation and identify the speaker behaviours contributing to these. Within the field of conversation therapy, SPPARC and BCA are positioned as ‘interaction‐focused therapies’ whose stated aim is to change the ‘behaviours used by the person with aphasia and/or significant other to deal with the impact of aphasic impairments on conversation’ (Wilkinson 2010: 54). The behaviours targeted for change in BCA are those acting as ‘barriers’ or ‘facilitators’ to conversation. Barrier behaviours function to limit PWA contributions; disrupt conversational flow; restrict the naturalness of conversation; lead to confusion and highlight linguistic errors or otherwise challenge the competence of the PWA. Examples include interruptions, asking ‘test questions’ where the answer is already known, and cueing to elicit ‘correct production’ of words or phrases (Beeke *et al*. 2013a, 2015). Meanwhile, facilitators incorporate CP behaviours that support the effective and natural involvement of PWA in conversation, such as allowing extra time, or the strategic use of passing turns and paraphrases (Lock *et al*. 2001). They also include PWA strategies that minimize the interactional impact of aphasia, e.g., employing total communication strategies, or strategically using keywords to set the topic (Beeke *et al*. 2015). BCA's primary outcome measure is the frequency with which barrier and facilitator behaviours are used in conversations before and after therapy (Beeke *et al*. 2015). Successful change is expected to be represented by a decrease in barrier behaviours and an increase in facilitator behaviours.

**Figure 1:**
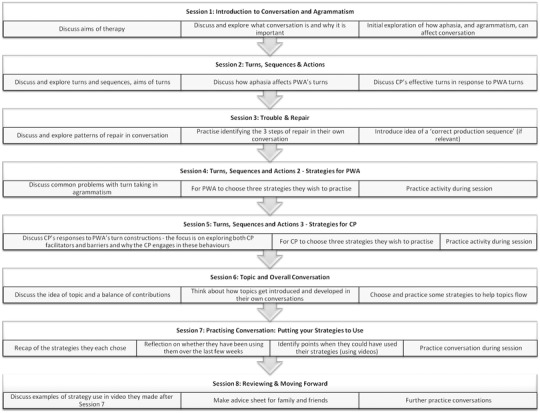
Better Conversations with Aphasia: the therapy programme.

BCA consists of eight sessions, to be delivered once a week jointly to the PWA and CP. The aims of each session are provided in figure 1.

The initial phase of therapy offers information about aphasia and conversation, with additional information on agrammatism as per the project's original focus. Videos of the dyad's own conversation are viewed in order to help speakers identify problem areas and existing strategies. Each partner then chooses a set of facilitative strategies to develop for use in conversation. Conversational strategy use is targeted by (1) reviewing video clips of problems in conversation and identifying strategies for dealing with them, (2) regular practice, both in open conversation and in more structured activities, and (3) experimenting with strategies between sessions and reflecting on the experience using a handout. For the full intervention programme, see the BCA online resource (Beeke *et al*. 2013b).

Existing literature on interaction‐focused therapies suggests that the mechanism responsible for enabling change is that of raising speakers’ awareness of their own conversational patterns (e.g., Wilkinson 2010). However, the assumption that increased awareness about a relevant behaviour leads to change in usage is challenged by accumulated evidence in other fields, which consistently show that developing knowledge about behaviour is not in itself effective for triggering behaviour change (e.g., Kennedy *et al*. 2004).

Interaction‐focused therapies also cite a broader ‘theory of change’. Kolb's experiential learning theory proposes that adult learners are best supported to develop and apply new ideas by reflecting on their previous experiences (Kolb 1984). This principle underpins the use of video, and the analysis of dyads’ own conversation patterns. However, Kolb's theory is concerned with describing knowledge creation, not behaviour change, and it does not position itself as a way of accounting for, or predicting, specific outcomes. A BCA case study by Beckley *et al*. (2013) illustrates how experiential learning principles successfully support one PWA to *recognize* his own conversational behaviour during therapy, but highlight that this speaker did not demonstrate evidence of independent behavioural change after therapy. So, while Kolb's model may offer insights into how a new awareness about conversation is developed, it does not provide a true theoretical account of BCA that is able to generate hypotheses about how the therapy leads to a change in the frequency of barrier and facilitator behaviour.

### Behaviour change theory

In order to develop a better‐matched theoretical explanation for how BCA leads to change in the use of barrier and facilitator behaviours, this study turns to behaviour change theory. The importance of understanding behaviour change within the field of rehabilitation is emphasized by Wade (2005: 812) who proposes that ‘all rehabilitation, at its heart, concerns changing behaviour’. A concerted research effort in health psychology is underway to develop the ‘science’ of behaviour change, and distil decades of accumulated evidence and theoretical knowledge into useable tools for designing, reporting and evaluating intervention (cf. Michie *et al*. 2014).

**Figure 2:**
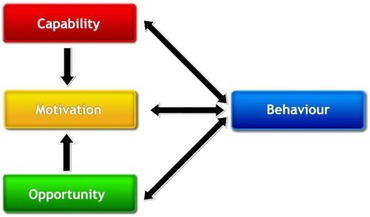
COM‐B Model of Behaviour (Michie *et al*. 2011).

A definition of ‘behaviour’ has been formulated as ‘Anything a person does in response to internal or external events … behaviours are physical events that occur in the body and are controlled by the brain’ (Michie *et al*. 2014: 234). Formal theories of behaviour are synthesized into the ‘COM‐B model’, a system for understanding behaviour that is placed at the heart of a wider framework for planning behaviour change interventions, called the ‘Behaviour Change Wheel’ (Michie *et al*. 2011). The COM‐B model positions individual behaviour in context as a product of three necessary conditions: the capability to carry out the behaviour, and the opportunity and motivation to do so (figure 2).

The condition of capability encompasses the knowledge and skills needed to carry out a specific behaviour. Knowledge incorporates awareness of the behaviour and its contexts, while skills incorporate the physical performance of the behaviour, the social skills needed to negotiate the behaviour's use, and the cognitive skills, such as memory, self‐monitoring and self‐regulation, which support an individual to initiate the right behaviour at the right moment. If framed within the context of the familiar International Classification of Functioning, Disability and Health (ICF) (World Health Organization (WHO) 2001), a person's capability to perform a particular behaviour can be understood as being determined not only by their trainable skills and knowledge, but also by the nature and severity and of their physical and cognitive impairments. opportunity encompasses any aspects of the external environment that enable or constrain the performance of a behaviour. Similar to the ICF construct of ‘environmental factors’ (WHO 2001) this includes both social and physical influences, e.g., the actions of others, or the availability of necessary resources. Finally, motivation refers to all ‘brain processes’ that ‘energise and direct behaviour’ (Michie *et al*. 2011: 4). motivation therefore covers the psychological determinants of behaviour, such as a person's consciously held goals and priorities—to which they direct their behaviour—and their beliefs about how well particular behaviours will serve these goals. It also includes an individual's confidence in carrying out behaviours effectively despite potential obstacles—known in the literature as self‐efficacy (Bandura 1997). Less‐conscious influences on behaviour are also incorporated in motivation, e.g., a person's identity, habits, outlook and emotions.

In seeking to investigate mechanisms of change in BCA, this study therefore aims to identify the changes in individual opportunity, capability or motivation that occur as a result of therapy, and which speakers report have played a role in changing their use of barrier and facilitator behaviour. The research question guiding this qualitative study is: According to BCA participants, how and why did their conversational behaviour change?

The analytic objectives of the study are:

- To identify the range of mechanisms reported by participants which support conversational behaviour change.
- To consider similarities and differences in how change is achieved for different types of behaviour (barrier and facilitator).

## Methods

Data and participants are drawn from the original BCA evaluation project, which was awarded multi‐site NHS ethical approval from Cambridgeshire 1 Research Ethics Committee (08/H0304/40). Participants were recruited via contact with aphasia support groups, University aphasia clinics and speech and language therapists (SLTs) working in the NHS and privately. Nine conversational dyads originally took part in the BCA therapy programme, 18 participants in total. A dyad usually consisted of a speaker with aphasia and their spouse; however, for some the main CP was a family member. Participants’ involvement in the study lasted 6 months, with pre‐ and post‐assessment phases, and the therapy programme itself each lasting for 8 weeks. See Beeke *et al*. (2015) for project design details.

### Participants

Data from eight PWA and eight CPs are used in the current study. One of the original nine dyads was excluded on the basis that they did not consent to the use of their data beyond the original project. Participants’ background details are provided in table 1.

**Table 1:** Details of participants

Dyad number and PWA pseudonym	Person with aphasia (PWA) age at recruitment (years)	PWA previous employment	Months since onset of aphasia (at time of first session)	Spoken word to picture matching (out of 40)^a^	Naming objects (averaged across three time points; out of 10)^b^	Conversation partner (CP) pseudonym and relation to PWA	CP age at recruitment (years)	CP employment
1: Kate	49	Jazz singer	33	38	8.33	Shelley (twin)	49	Publishing
2: Simon	39	Own business	30	36	8.33	Cath (wife)	Late 30s	Runs day nursery
3: Giles	55	Senior sales manager	59	39	9.00	Linda (wife)	Mid‐50s	Information technology operations manager
4: Graham	63	Hospital manager	60	35	3.33	Alex (partner)	Early 60s	A&E nurse (retired)
5: Jill	57	Cashier at bookmakers	39	24	5.33	David (son)	30s	Own business (trading)
6: Barry	60	Gardener/book illustrator	17	39	4.00	Louise (wife)	Early 60s	Housewife
7: Maggie	71	Deputy head teacher	40	35	4.00	Christina (daughter)	Late 30s	Artist
9: Bob	67	Graphic designer and musician	48	29	1.33	Irene (wife)	60s	Office worker (retired)

Notes: ^a^PALPA47 (Kay *et al*. 1992).

^b^An object and naming battery (Druks and Masterson 2000).

### Data

The data consist of samples from video recordings of BCA therapy sessions (during‐therapy data), and audio‐recorded interviews with the participants about their experience of therapy carried out 6–24 months post‐project (post‐therapy data). Transcripts of the data feature the acronyms PWA, CP, SLT and R (researcher).

### During‐therapy data

These data consist of four discussion‐based activities occurring within therapy in which participants report back to the treating SLT on their attempts to make behavioural changes within everyday conversations at home. The discussion is focused on the experience of the PWA in session 5, and of the CP in session 6. In session 7, two discussions take place involving both speakers. The first reviews the dyad's attempt to make changes since the last session, whilst the second focuses on whether participants have been using their chosen strategies on a routine basis, and if not, why not. The length of these discussions varied greatly from session to session and from dyad to dyad. They typically lasted 6–8 min; however, they could range from 2 to 22 min. The video recordings of these activities were transcribed verbatim by the first author. For the sake of efficiency, transcription omitted any long asides that were not directly relevant to conversational behaviour.

Unlike research interviews—which take a systematic approach to probing the underlying meaning of participant reports, and avoid leading questions or comments—these discussions were informal explorations of the participants’ experience of making changes, which were also guided by the need to provide therapeutic advice and feedback. Direct confirmation or elaboration from the PWA was sometimes sought out, but not as part of a systematic strategy. Implications for data analysis are discussed below.

### Post‐therapy data

Post‐therapy data consist of semi‐structured research interviews which were designed and carried out by the first author as part of the BCA evaluation project. Questions were developed primarily to gather feedback about participants’ perception of BCA and their self‐reported outcomes. However, additional questions focused on the experience of attempting change during therapy, such as ‘What helped make therapy successful/what stopped it being successful?’ and ‘What was your role in making the therapy work?’.

Participants had not met the first author during their involvement in the project, and were encouraged to provide honest feedback. Interviews were initially intended to be carried out separately with the PWA and CP, with final questions for joint discussion. However, not all PWA wanted to be interviewed alone. In the end only PWAs 2, 5 and 7 were interviewed separately.

The interview was carried out in accordance with the questioning style recommended for typical research interviews, i.e., open‐ended questions, and avoidance of interpretive comments (Britten 1995). However, specific modifications to this approach were employed in order to maximize PWA contributions, as recommended by Luck and Rose (2007). This included offering a closed choice of possible responses to PWA where needed, and interpretively paraphrasing PWA contributions in order to confirm the interviewer's understanding of what has been said. Every effort was made to corroborate and elicit views from the PWA directly; however, it should be acknowledged that the success of these techniques was variable among the most impaired participants. Interviews typically lasted for about 1 h, with the shortest lasting 47 min and the longest 75 min. They were audio‐recorded and transcribed in full by the first author.

Table 2 summarizes the quantity of transcribed data prepared for analysis. The average number of pages of transcribed data per dyad is 17 (median), with a range from 12 to 22 pages.

**Table 2:** Transcribed data for analysis per dyad and data source

	Pages of transcribed data		
Dyad number	During therapy	Post‐therapy	Total pages
1	8	10	18
2	5	12	17
3	7	15	22
4	8	8	16
5	6	12	18
6	3	9	12
7	7	8	15
9	4	13	17
Total pages	48	87	166

### Analysis

**Figure 3:**
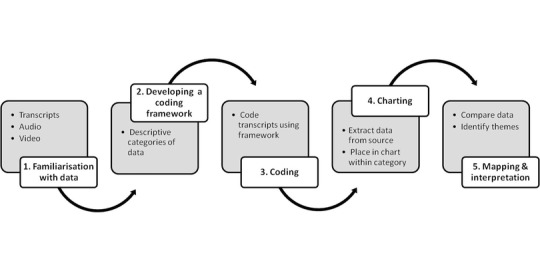
Five‐step process in Framework Analysis, based on Ritchie and Spencer (1994).

Data were analysed using Framework Analysis (Ritchie and Spencer 1994), a method developed by the National Centre of Social Research (www.natcen.ac.uk) to support the needs of applied research. The process of analysis follows five steps (figure 3).

Step 1 involves listening to data and reading transcripts, whilst noting any recurring themes. Step 2 is the process of designing and testing a coding framework to capture data with relevance to the research questions. At this stage coding categories are pragmatic, rather than interpretive—so, for example, the deliberately broad label ‘Factors supporting behaviour change’ was developed to identify relevant content in the transcripts. In step 3, all transcripts are coded; in step 4 coded portions of text are extracted from the transcripts and collated together in a chart. The final step—mapping and interpretation—represents the inductive process of thematic comparison and analysis common to many qualitative approaches. Here, all items of data collected within coding category are compared against each other for similarity and difference. The researcher is looking to group items that share features and which may represent a similar phenomenon, e.g., the same underlying reason for changing one's behaviour. Conceptually similar items are brought together under a descriptive ‘theme’ able to represent their commonality. These themes aim to represent and explain the full range of meaning in the dataset and are a core analytic output of Framework Analysis. Where unusual items of data do not ‘fit’ into the emerging themes, all data must be re‐examined and, if appropriate, new thematic explanations drawn up. In some cases such items of data may be judged to be conceptually distinct enough from the rest of the dataset to warrant their own analytic category. Themes may therefore represent a large number of data items, or just one or two.

In this study, the coding process (step 3) sought to counter any influence on PWA data from the contributions of others. Data were therefore only coded when they clearly represented a speaker's own account of their behaviour. This meant that CP and SLT comments or speculations about PWA behaviour were excluded from the analysis, as were PWA contributions whose meaning remained ambiguous, even despite verification. During step 4, NViVo data management software was used to compile the coded portions of text into charts. Step 5 sought to develop sufficient analytic themes to describe the full range and the nature of the factors that participants had reported as supporting change via therapy, i.e., BCA's mechanisms of change. The process of analysis was iterative, and moved back and forth between the quotes, the descriptive themes emerging, and the theoretical concepts represented within the COM‐B model, in order to develop the most accurate and theoretically useful account of the data. As an example, consider the following quote, in which CP3 discusses actively withholding support in order to promote PWA verbal output:

Before we had that therapy, we'd sort of let you try and work out, try and tell us things … even though when we knew perhaps what you were going to be saying, we wanted you to say it because we thought it was helpful.

*Post‐therapy: CP3*

Here, CP3's termination of this barrier behaviour is understood as being attributed to a change in perception about its overall ‘helpfulness’. Similar shifts in perception were observed throughout the data. Brought together, these data were interpreted as reflecting underlying changes in speakers’ ‘beliefs about the consequences’ of their behaviour: a theoretical concept associated with motivation (Michie *et al*. 2014). The thematic description *Changed expectation of behaviour's impact* was therefore developed to represent these data, and offer a theoretically coherent reflection of changed motivation.

## Results

Seven themes were identified in the data, each representing a potential mechanism of conversational behaviour change. These are presented in table 3, alongside references to the speaker from whom the source data originate.

**Table 3:** Mechanisms of conversational behaviour change identified in the data

			Data
opportunity	Mechanism 1	*Change in conversational support for PWA strategies*	PWAs 5, 6; CP5
capability	Mechanism 2	*Increased awareness of own behaviour*	CPs 3, 5–7
	Mechanism 3	*Replacing barriers with facilitators*	CPs 1, 3–5, 7
	Mechanism 4	*Increased ease at implementing strategies*	PWA2; CPs 2, 4
motivation	Mechanism 5	*Changed expectation of behaviour's impact*	PWAs 2, 6; CPs 1–6
	Mechanism 6	*Changed priorities for conversation*	CPs 3, 5, 6
	Mechanism 7	*Changed perception of success in conversation*	CPs 6,7

The following discussion of mechanisms and accompanying data is placed within the context of the COM‐B model. Individual mechanisms are illustrated using key quotes chosen to represent the thematic qualities of the analysed data. However, the description of each mechanism is based on all items of data.

### Changing opportunity to change behaviour

opportunity represents all influences external to an individual which determine the behaviours they use. Mechanisms for changing behaviour via opportunity therefore entail any environmental change that triggers the use of a new behaviour, or prevents the use of a habitual one. Analysis of data revealed one change in conversational opportunity impacting on improved strategy use, presented here as BCA's mechanism 1.

#### Mechanism 1: Change in conversational support for PWA strategies

A core assumption underpinning the BCA approach is that PWA will use strategies more successfully in conversation if CPs offer appropriate scaffolding and support. Evidence from two dyads (D5, 6) confirms this. The data from dyad 5 below suggest that the PWA's use of conversational writing only occurs when prompted by the CP:

CP: Being honest it's the writing things down that, that's our fallback.

R: That's interesting, as you [i.e., PWA] were saying—yeah, we worked on writing, but I don't use it that much these days. So you need David to give you that reminder?

CP: Give you a kick up the bum!

(laughter)

R: You wouldn't pick up a pen.

PWA: Yeah

CP: No if my mum comes to my house, that's when, she gets a pen and paper in her hand.

*Post‐therapy: D5*

The role of CP in enabling PWA strategy use is further illustrated by data showing that CP5 also supports PWA5's writing by making pen and paper available in different environments, and—in dyad 6—from PWA6's feedback that that CP6 allows him more time for his conversational turns. Together, these data confirm that the changes made by CPs after therapy directly impact on PWA behaviour. They also demonstrate that increasing the conversational opportunities available for PWA strategy use is one way in which BCA operates to produce behavioural change among PWA.

### Changing capability to change behaviour

In the COM‐B model, capability represents the ways in which a person's knowledge and skills determine their use and effective performance of a behaviour. Behavioural changes brought about by changes in capability may therefore arise from an increased awareness about a behaviour, and/or an increased skill for negotiating its use or performing it in the right way at the right moment. The current analysis identified three potential changes to capability linked to evidence of conversational behaviour change.

#### Mechanism 2: Increased awareness of own behaviour

Several CPs report a change in what they frequently refer to as an ‘awareness’ of their own conversational behaviour, echoing the therapeutic mechanism previously proposed by the SPPARC and BCA literature (Beckley *et al*. 2013, Lock *et al*. 2001). The data presented here help to further specify this concept, and demonstrate how it interacts with speakers’ beliefs about behaviour to activate change.

For CP6 and CP7, becoming aware simply means observing a behaviour of interest within their own communication—as illustrated below with the example of passing turns:

CP: I'll tell you what I did notice, and this was when I got back. I think I had to pay for something on the phone, and I caught myself going ‘mm’, so I realise I do do it.

SLT: Yeah, everyone does

CP: Yeah, but you just don't realise you do it. But I was aware of it then.

During therapy: CP7

While the reports from CP6 and CP7 confirm that BCA can support participants to recognize certain behaviour, these remarks are not overtly linked to an attempt to use the behaviour more frequently or more strategically. It is therefore unclear that increased recognition has a direct relationship to prompting behavioural change.

In a different type of CP account, increased awareness involves more of a judgement about the value of the behaviour used—either in terms of the costs of using barriers (CP3, CP5) or the benefits of using facilitators (CP7). Unlike the reports of merely observing a behaviour, these evaluative accounts are linked in the data to active attempts to change behaviour. In the quote below, CP3 reports a newfound awareness of her behaviour of withholding support, but also of its negative impact (i.e., her partner struggling):

I've been giving you words rather than letting you struggle. Things like that. Cause I think that has—that's made me much more aware of what I was doing. Before.

*During therapy: CP3*

For CP3, and the other speakers providing this type of account, making a change in conversation is attributed not just to an increased recognition of their own conversational behaviour but in particular to an increased insight into the consequences of that behaviour for the conversation, or for their partner. While these data confirm the hypothesis that BCA can work to increase participants’ awareness of their conversational behaviour, they also suggest that for this process to be an active mechanism for change, it needs to include an explicit evaluation of how that behaviour impacts on conversation. Simply being able to recognize an example of one's own behaviour may not be sufficient.

#### Mechanism 3: Replacing barriers with facilitators

Many CPs worked simultaneously on learning to inhibit barrier behaviour whilst also developing new uses of facilitators. There is evidence in the data that *linking* these two processes supported CPs to make changes in context. CPs describe explicitly using a facilitator *instead of* a barrier, for example: CP3 giving extra time instead of interrupting or CP4 paraphrasing PWA4 instead of saying ‘I don't understand’.

The quote below illustrates how this conscious self‐regulatory activity is experienced by speakers. Here CP1 talks about PWA1 producing a word (‘man’) where the context is not clear. Instead of using her habitual strategy of rapid questioning to establish PWA1's meaning, CP1 attempts to leave space and use passing turns:

You think ‘What's this?’ Y'know. And then it was—I just knew, and all I did was my I—which was to listen—and let Kate carry on, rather than going ‘Yeh? Man? What about man? Which man? What man, where?’ I thought ‘Right: I'm gonna shut up and not say anything, so yep—listen listen’.

*During therapy: CP1*

The inner dialogue reported by CP1 illustrates the conscious effort she is directing towards inhibiting a previous behaviour and activating an alternative. The data gathered under this theme suggest that making a behavioural change during conversation is supported by an internal self‐regulatory process, in which speakers cue their own strategy use by noticing moments where they would habitually use a barrier. As a mechanism for supporting change, the act of ‘replacing’ therefore offers benefits for clarifying and simplifying the effort involved in initiating a change at the right moment in conversation.

#### Mechanism 4: Increased ease at implementing strategies

Several CPs and PWA reported an increasing ‘ease’ at using facilitative strategies in conversation. These data suggest that the skill for implementing strategies may improve over the course of therapy, as illustrated by quotes from different time points and different types of speaker:

And also how to support you when you're talking innit. The prompts the aids and all that. Which you just start to use easier.

*Post‐therapy: CP4*

SLT: And how easy are you finding it to think. You know if the conversation is stopping, or you're having difficulty getting a word out. How easy are you finding it to sort of switch into doing something else?

PWA: Yes. It's alright.

CP: I think you're thinking about that a lot. When you're talking.

PWA: Yeah. Yeah.

SLT: Is it getting easier, or are you having to think about it a lot?

PWA: Um. Getting on, getting on. Getting better.

*During therapy: PWA2*

The data gathered under this theme indicate that BCA can reduce effort for strategy use in conversation, thereby supporting longer term behavioural change. However, the exact nature of the skill change represented by this mechanism is unclear from these data. Successful strategy use in conversation is likely to depend on a speaker's practical skills for executing a behaviour such as writing or paraphrasing, but also on their wider cognitive skills, e.g., remembering to use a new behaviour, and recognizing when to do so. It seems plausible that BCA may be strengthening either or both of these areas in order to change participants’ experience of ease for strategy use.

### Changing motivation to change behaviour

motivation within the COM‐B model represents a complex collection of behavioural determinants, including a person's beliefs, priorities, and self‐efficacy. Behavioural change driven by changes to motivation may therefore involve a change in people's beliefs about the impact of their behaviour, a change to what it is they want to achieve by their actions, or a change to their confidence in performing and persevering with a behaviour. Evidence from this analysis indicates three potential changes in motivation that may be relevant to conversational behaviour change.

#### Mechanism 5: Changed expectation of behaviour's impact

Both PWA and CPs discussed how their beliefs about the costs and benefits of specific behaviours evolved during therapy, with speakers often explicitly attributing their behavioural changes to this process. The increased use of facilitators was linked to strengthened expectations that a behaviour would benefit conversation, whilst the reduction in barriers appeared to be triggered by the realization that some behaviours had negative impacts. These are discussed in turn.

Following a period of experimentation with target facilitators, CPs reported observing benefits associated with the strategies, including furthering the level of understanding between speakers (CPs 1, 4–6), improving the naturalness of conversational dynamics and atmosphere (CPs 1, 2, 6), reducing frustration and worry (CPs 4, 6) and generalized benefits for the dyad's relationship (CP4). There is some evidence that this process is relevant for PWA too (PWAs 2, 6). The extract below illustrates the change in attitude experienced by PWA6 during therapy, in relation to using pen and paper in conversation:

R: So it's about you making sure you've got paper and pens?

PWA: Yes, yes. Good, I think yes.

PWA: I go ‘ooh’ it's … (grimacing facial expression)

R: Don't wanna do it

PWA: Yeah but no I think yeh, yeh. Good.

R: So at first you were like, oh, um, dunno

PWA: Yeah. But no I think it's … oh.

*Post‐therapy: PWA6*

The data in this theme suggest that positive expectations about the outcome of using facilitative strategies can be built up during therapy, and that this may act as a mechanism for promoting ongoing use.

Changing speakers’ expectations about the impact of barrier behaviour appears to be directly linked to changing usage. CPs often reported changing their use of barrier behaviour after having identified the costs for the dynamics of the conversation (CPs 4–6) or for the emotions of the PWA (CP4). The following extract is representative of this process:

I would ask questions that I would already know the answer to, y'know. So. I was aware I was doing it, but I wasn't aware of how it was affecting our conversation. So that definitely opened my eyes a bit. And helped me. And obviously those things, for myself. They've stayed with me. I became aware of them over the few months that we were doing the therapy. Once you break a habit.

*Post‐therapy: CP5*

The quote highlights the point first raised in relation to mechanism 2, i.e., that having an awareness of one's own behaviour may not be sufficient for change until the consequences of that behaviour have been evaluated. The realization that one of their behaviours had a negative impact had the potential to resonate very strongly. Both CP4 and CP5 reported that this was the main thing that they remembered about therapy. BCA's ability to produce a shift in perception about the impact of behaviours appears to be a key motivator for change for both PWA and CPs, and across both categories of behaviour (facilitator and barrier).

### Mechanism 6: Changed priorities for conversation

Prior to BCA, a number of CPs were observed engaging in behaviours designed to pursue accurate verbal output from the PWA, often regardless of whether the PWA had already successfully communicated their intended meaning. These CPs reported feeling a responsibility to promote accurate speech within conversation with the expectation that this would in some way be valuable for their partner. Among some of these speakers, there is evidence that participating in BCA prompted a re‐evaluation of the priority placed on accurate speech in conversation, and a shift towards accepting any mode of successful communication instead. Furthermore, there is evidence that this change in priorities was linked to reducing barrier behaviours such as test questions or correct production elicitations. The quote below from CP6 illustrates this shift in attitude, and its potential to support behavioural change:

CP: I think that's what came out of it. Instead of concentrating on oh Barry MUST speak, we must do this, we must do that. No. Communicate!

PWA: Yeh

CP: Y'know whichever way. Gestures. Writing. I think that was the biggest thing. Don't worry about it so much, as long as you communicate. Whichever way.

*Post‐therapy: CP6*

These data suggest that BCA has the potential to prompt change in the use of barrier behaviours directed at eliciting accurate verbal communication by supporting speakers to re‐evaluate their beliefs about what conversation is for, and to reconsider how helpful and effective those specific barrier behaviours really are.

#### Mechanism 7: Changed perception of success in conversation

Data in this theme suggest that BCA has the potential to enhance positive perceptions among CPs about their own abilities as a CP to someone with aphasia, thereby suggesting BCA can play a role in promoting speaker self‐efficacy. The following quote from CP7 illustrates the potential benefits of identifying pre‐existing facilitative behaviour in conversation in terms of boosting self‐confidence in one's own skills:

CP: You kinda think well, do we ever have conversation? And it made me think: I don't think we have much conversation. But we do. And we did. Particularly when we sat down and did the videos—obviously sometimes it was quite difficult but other times it was quite natural wasn't it?

PWA: Yup

CP: And it just showed. We were doing some things that were right. We worked our way round it. The communication problems.

*Post‐therapy: CP7*

The therapeutic process reflected here focuses on the recognition of success in existing strategies, rather than practicing new strategies. As in mechanism 2, the process of ‘recognition’ is not specifically linked in the data to future attempts by participants to use the identified behaviours. It is therefore unclear if an enhanced perception of success in conversation truly works as a direct mechanism for changing the way in which pre‐existing facilitators are used. It may be that the strengthened self‐efficacy indicated here represents a distinct outcome of the BCA programme in and of itself.

### Comparing mechanisms involved in change for barriers and facilitators

**Figure 4:**
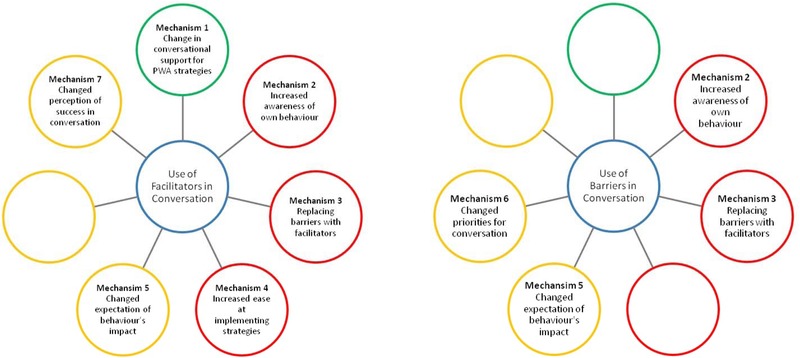
Comparing mechanisms supporting change in the use of barrier and facilitator behaviours

The mechanisms associated with changing barriers and facilitators are compared in figure 4, which shows that not all mechanisms hold equal relevance for barriers and facilitators.

Reducing or completely terminating barrier behaviour relies on a combination of mechanism 2 and mechanism 5 in order to motivate change, with data suggesting that therapeutically orchestrated ‘realizations’ about the negative impact of barriers may be particularly powerful. The motivation for change may also arise from mechanism 6, a broad shift in priorities for conversation away from accurate verbal production and towards effective communication and social rapport. The capability to terminate these habitual behaviours appears to be supported by mechanism 3, i.e., using a replacement behaviour.

While a combination of these four mechanisms may be sufficient for prompting change in barrier behaviour, the process of embedding facilitative strategies into everyday conversation appears to be more complex, with more potential for variation—involving combinations of up to six mechanisms. For PWA, increasing opportunity for facilitator use, via mechanism 1, may act as the core mechanism for producing behavioural change. Indeed, Beckley *et al*.’s (2013) case study of PWA3 suggests that where self‐initiated behaviour change may be limited by difficulties in comprehension or cognition, new PWA strategy use may be entirely dependent on the prompts and opportunities provided by CPs. The results also highlight subtle differences between the mechanisms engaged for pre‐existing facilitators and new facilitators. Mechanisms 2 and 7 are primarily relevant to the identification and evaluation of facilitative behaviour already used by speakers. These mechanisms promote speaker self‐efficacy for conversation with aphasia and link this to specific strategies. Strong evidence in other fields suggests that self‐efficacy is a good predictor of behaviour and an effective mechanism for motivating behavioural change (Bandura 1997). However, the current data do not clearly link raised awareness and self‐efficacy to the future strategic use of the behaviours under discussion. This raises the possibility that the targeting of self‐efficacy in BCA could be further optimized in order to maximize change. Meanwhile, BCA's structured practice and review of new facilitators contributes to mechanisms 3–5. This process relies on initial experimentation with strategies and identification of their benefits, thereby providing a motivation for future use. Repeated practice subsequently supports the capability for sustaining strategy use, by developing internal cues about when to initiate strategies, and by increasing ease of implementation.

The finding that barriers and facilitators engage overlapping, but ultimately different, processes of change represents a new insight in conversation therapy, and will be important for understanding and evaluating BCA—and similar interventions—in future research. The relative strength, importance, or universality of these mechanisms for creating change to barriers and facilitators is not possible to deduce from these data. However, existing outcomes from the BCA project suggest that therapy may be more consistently effective at reducing barriers than embedding facilitators (Beeke *et al*. 2014, 2015). With many CPs showing a significant reduction in barrier behaviours, it seems possible that the mechanisms described here may be both effective and sufficient for barrier change among these speakers. The pattern of change in facilitator use among CPs and PWA is more variable. Explanations for this include likely difficulties in capturing change to pre‐existing facilitators, as well as the possibility that the mechanisms and therapy content targeting facilitator use need to be further optimized. However, the finding here that the change process for facilitators involves more variation and complexity than for barriers also offers a new perspective on the challenges of embedding strategy use in conversation.

## Discussion

This study offers the first systematic, data‐driven and theoretically grounded account of how a conversation therapy for aphasia may produce change. Central to this analysis is the proposition that the primary outcome for BCA—and other conversation therapies—is one of behaviour change. The conversational behaviour changes targeted by BCA include the active inhibition of barrier behaviours and/or the active adoption, or redirection, of facilitative behaviours—in order to manage strategically the conversational problems caused by aphasia. The findings presented here illustrate a complex process of behaviour change that has not previously been adequately represented by the emphasis on ‘raised awareness’. While this study demonstrates that an *Increased awareness of own behaviour* (mechanism 2) is indeed a component of the change process set in motion by BCA, it also shows that this is only one of several interacting mechanisms promoting conversational behaviour change. Crucially, this study suggests that raised awareness of one's own behaviour is not in itself sufficient for change, and instead that speakers should also evaluate the *impact* of their behaviour.

By combining the principles of behaviour change theory with a qualitative investigation of participants’ own explanations of behaviour change, this study concludes that BCA operates by strengthening speakers’ reasons to do something differently (motivation), and by structuring and supporting their efforts to make changes in context (capability). BCA also offers the potential to *indirectly* increase strategy use among PWA who are unable to activate self‐regulated change by changing the conversational support offered by CPs (opportunity). This reflects BCA's roots in CP training programmes, where indirect environmental change has long been proposed to be a mechanism for revealing PWA competence (Kagan and Gailey 1993).

The study offers the key finding that ‘stopping’ a conversational behaviour (i.e., a barrier) will involve a different process of change than that involved in ‘starting’ to use a new behaviour, or extending an existing behaviour (i.e., a facilitator). Reducing or terminating behaviour relies on changing speakers’ beliefs about how the behaviour functions in conversation, and on shifting their priorities from accuracy to interactive efficiency and naturalness.

The process used in interaction‐focused therapies to target facilitators is influenced by experiential learning theory and consequently involves active experimentation with strategies, followed by self‐reflection (Beckley *et al*. 2013, Lock *et al*. 2001). The findings here confirm that experimentation with and reflection on facilitator use contributes to change. However, they show we can be much more detailed about *how* experimentation and reflection work to promote change. Specifically, it is suggested that initial experimentation with strategies provide a focus for speakers to reflect on the *impacts* of using facilitators. Identifying the benefits of a specific behaviour for conversation has the potential to change people's minds about the value of the strategy, and build up positive beliefs which motivate further use. In addition to an initial phase of experimentation, this study has highlighted the role of repeated practice of strategies for increasing ease of use. This reflects the literature on habit‐formation, which proposes that ‘automaticity’ in behaviour is built up through frequent experiences of producing the behaviour in service of a specific goal (Aarts and Dijksterhuis 2000). Sociocultural theories of learning also emphasize the role of repetition, not just for promoting automaticity in new skills, but also for the ability to use those skills flexibly and creatively in complex, interactive, tasks (Hengst *et al*. 2010). Conversational strategy‐training therefore needs to consider not only how to structure initial experiences of successful strategy use, but also how to create repeated opportunities for speakers to use new strategies in conversation.

One mechanism of particular interest, with relevance to both stopping old behaviours, and activating new ones, is mechanism 3: *Replacing barriers with facilitators*. The value of using one behaviour to cue another is evidenced in Simmons‐Mackie *et al*. (2005), who show a dramatic drop in one CP's use of test questions *only* after the speaker was explicitly provided with an alternative behaviour. This process is also supported by a Lustig and Tompkins (2002) case study, in which a PWA is trained to recognize articulatory struggle in conversation as a cue to pick up a pen and try writing. These findings tie in with the psychological literature on ‘implementation intentions’: a form of action plan which explicitly links the performance of a target behaviour to a specific contextual cue, i.e.,: ‘if X happens then I will do Y’ (Gollwitzer and Sheeran 2006). Cues can be environmental, or they can be internal, e.g., a thought or feeling. A key meta‐analysis from Gollwitzer and Sheeran (2006) of both published and unpublished investigations demonstrates that implementation intentions have a medium to large effect on behavioural change. Furthermore, this effect size is even larger among populations with impaired self‐regulation, including those with brain injuries. This literature provides important support for the role of mechanism 3 in supporting behavioural change. Explicit consideration of how speakers can be supported to remember and regulate changes during the activity of conversation is relatively rare in the therapy literature, so this finding represents a promising new avenue.

In the last of its key findings, this study suggests that the process underpinning change in the use of pre‐existing strategies may be slightly different to that underpinning the introduction of new strategies. Simmons‐Mackie and Damico (1997) propose that treatment for compensatory communication strategies will have the most success when seeking to extend existing behaviour for new strategic purposes. Exploring the most effective mechanisms for supporting new and extended uses of pre‐existing facilitators will therefore be an important future direction for research. It will also be important to question whether looking for an increased *frequency* in existing behaviour is the right way to evaluate the change that the therapy process is addressing. It may be that changes in the use of pre‐existing behaviour will be better represented—and more easily demonstrated—by looking at when, how and why these behaviours are used.

In summary, this study has yielded clinically useful and important insights into how BCA may operate to create change in conversation. Although the BCA project originally targeted people with agrammatic aphasia, the relevance of the mechanisms identified here may extend to interaction therapies for other populations. Exploring such applications may represent a useful aim for future research. It should be noted, however, that identifying evidence for the presence of these mechanisms of change does not equate to evidence for their effectiveness. The relative priority or strength of the identified mechanisms of change remains unclear, and it is unknown if all mechanisms apply to all speakers. This study's exploratory focus means its findings have not been analysed in the context of participants’ objectively measured change. Future research is therefore needed to evaluate the relationship of reported mechanisms to BCA's actual behavioural outcomes. In addition, the account of change developed in the current study lacks crucial information about the ingredients of therapy that may be responsible for activating the mechanisms reported here. This is addressed in a companion piece to this paper which focuses on identifying and describing BCA's active ingredients for changing behaviour (Johnson *et al*. 2016).

It is recognized that this study faces certain limitations. The retrospective nature of the study design, and the relative wealth of data elicited from CPs compared with PWA mean the account of change developed here is unlikely to be comprehensive, or definitive. It is also recognized that self‐reported data about behaviour change will always be constrained by the limits of self‐knowledge, i.e., that we may not always truly know why we behave the way we do (Paulhus and Vazire 2007), and also by the difficulty in reliably reflecting on the higher order cognitive processes that regulate behaviour (Nisbett and Wilson 1977). It is certainly true that where participants describe experiences of ‘ease’, or ‘awareness’ we cannot be sure these terms are being used in the same way to describe the same thing. The conclusions drawn here about the involvement of specific cognitive skills in changing conversational behaviour need to be treated with caution, however the discussion of the psychological literature on habit formation and implementation intentions has offered some interesting indications as to how to understand these qualitative data.

While we argue here that behaviour change is the primary goal of intervention for conversation, it is nonetheless recognized that conversation therapy for aphasia is intended to support wider psychosocial adjustment and improved quality of life. The behavioural outcomes of BCA and the nature and extent of their relationship to changes in functional activity, social participation, quality of life and wellbeing needs to be further investigated in order to provide the most comprehensive account of therapy's effects.

## Conclusions

Exploring qualitative evidence for BCA's mechanisms of conversational behaviour change has generated concrete suggestions about therapy's core processes. Although preliminary, these findings offer guidance to clinicians wishing to replicate the therapy in novel situations, or to streamline it where time and resource constraints limit implementation of the full BCA programme. A key innovation of this study has been the use of theory and tools from the field of behaviour change to understand *how* therapy works. The application of a behaviour change perspective in speech and language therapy is expected to have wide‐ranging benefits, for both researchers seeking to develop and evaluate well‐justified interventions, and working SLTs, among whom designing therapy intervention is the cornerstone of clinical practice.
